# Clinical changes in symptomatic cervical ectopy following vaginal hyaluronic acid therapy

**DOI:** 10.3389/fmed.2026.1832997

**Published:** 2026-04-20

**Authors:** Orhan Yanar, Sadık Orhun Aktaş, Serap Simavlı, Yüksel Onaran, Ömer Hayri Yalınkaya

**Affiliations:** 1 Department of Obstetrics and Gynecology, The Nev Hospital, Şanlıurfa, Türkiye; 2 Department of Obstetrics and Gynecology, Etimesgut Martyr Sait Ertürk State Hospital, Ankara, Türkiye; 3 Department of Obstetrics and Gynecology, Üsküdar University, Istanbul, Türkiye; 4 Department of Obstetrics and Gynecology, Turkish Ministry of Health, Ankara Bilkent City Hospital, Ankara, Türkiye; 5 Department of Obstetrics and Gynecology, Dünyapark Hospital, Diyarbakır, Türkiye

**Keywords:** cervical ectopy, epithelial transformation, gynecology, hyaluronic acid, non-ablative therapy

## Abstract

**Background:**

Cervical ectopy is a common physiological condition in women of reproductive age and may be associated with symptoms such as vaginal discharge, postcoital bleeding, dyspareunia, and pelvic pain. While ablative treatments are frequently used in symptomatic cases, interest in non-ablative approaches has increased. This study evaluated clinical and epithelial changes following vaginal hyaluronic acid therapy in women with symptomatic cervical ectopy.

**Methods:**

This prospective observational study included 121 women diagnosed with symptomatic benign cervical ectopy. All participants received hyaluronic acid vaginal ovules (Cicatridine) once daily for 14 days. Follow-up was performed at 4 weeks, 8 weeks, and 3 months after treatment. Epithelial transformation was assessed by quantifying the extent of columnar epithelium during speculum examination. Temporal changes were analyzed using repeated-measures ANOVA.

**Results:**

The mean epithelial transformation rate increased progressively from 61.5% ± 19.8 at 4 weeks to 74.6% ± 21.2 at 8 weeks and 82.9% ± 20.4 at 3 months (*p* < 0.001). Clinical follow-up notes suggested a trend toward symptom relief; however, symptom change was recorded descriptively without a validated scoring instrument.

**Conclusion:**

Vaginal hyaluronic acid therapy was associated with progressive epithelial transformation in women with symptomatic cervical ectopy, with descriptively observed symptom relief. However, because of the observational design, absence of a control group, and non-standardized symptom assessment, these findings should be interpreted as exploratory. Further randomized controlled trials are required to determine clinical effectiveness.

## 1 Introduction

Cervical ectopy (also termed cervical ectropion) is a common benign gynecological finding characterized by the presence of endocervical columnar epithelium on the ectocervical surface due to eversion of the endocervical canal (1). It is frequently observed in reproductive-age women and is strongly influenced by hormonal status, particularly estrogen-related epithelial dynamics (1, 2). Although prevalence estimates vary across populations and settings, cervical ectopy is widely encountered in routine gynecologic practice (1, 2).

In many women, cervical ectopy remains asymptomatic; however, a subset presents with bothersome symptoms such as persistent vaginal discharge, postcoital bleeding, dyspareunia, and pelvic discomfort (1, 3). In symptomatic cases, management is primarily directed toward improving quality of life and controlling symptoms rather than treating a premalignant lesion (1).

Conventional treatment options for symptomatic ectopy are mainly ablative and include cryotherapy, electrocautery, and laser-based techniques, aiming to induce squamous metaplasia of exposed columnar epithelium (3, 4). Cryotherapy is among the most commonly used approaches and has shown symptom benefit in selected patients (3, 4). Nevertheless, ablative therapies may be associated with procedure-related discomfort and, in some contexts, complications such as cervical stenosis or scarring (5).

Interest has therefore increased in non-ablative strategies that support mucosal homeostasis and epithelial remodeling. Hyaluronic acid is a key extracellular matrix glycosaminoglycan with recognized roles in hydration, tissue integrity, inflammatory modulation, and epithelial repair dynamics (6, 7). These biological properties provide a rationale for its local use in cervicovaginal conditions. Topical hyaluronic acid formulations have been used to support cervical and vaginal mucosal recovery in clinical gynecology (8, 9). However, evidence specifically addressing symptomatic cervical ectopy remains limited compared with ablative interventions (3, 4). Accordingly, the present prospective observational study aimed to evaluate clinical and epithelial changes following vaginal hyaluronic acid ovule therapy in women with symptomatic benign cervical ectopy.

## 2 Materials and methods

### 2.1 Study design and setting

This prospective observational clinical study was conducted at the Department of Obstetrics and Gynecology, Bolu Abant İzzet Baysal University, Turkey. The protocol was approved by the Institutional Ethics Committee of Bolu Abant İzzet Baysal University (protocol code: 2012-011; approval date: 28 February 2022). All procedures followed the Declaration of Helsinki, and written informed consent was obtained from all participants before enrollment.

### 2.2 Study population

Women who presented to the gynecology outpatient clinic between January 2022 and December 2022 and were diagnosed with symptomatic benign cervical ectopy were evaluated for eligibility. A total of 121 women aged 24–41 years who met the study criteria were included.

Cervical ectopy was defined as the presence of columnar epithelium extending at least 1 cm beyond the external cervical os on speculum examination. This threshold was selected to ensure clinically visible and reproducible measurements.

The 1 cm threshold was used as an operational criterion for standardized follow-up in this observational cohort and should not be interpreted as a validated severity cutoff.

### 2.3 Inclusion and exclusion criteria

Eligible participants were women aged 24–41 years with symptomatic benign cervical ectopy and available baseline and follow-up data.

In this cohort, “symptomatic” status was defined as the presence of at least one bothersome patient-reported complaint (abundant leukorrhea, contact bleeding, dyspareunia, or pelvic pain) at presentation. No validated symptom severity scale was used; symptoms were recorded descriptively. Exclusion criteria were pregnancy; cervical intraepithelial neoplasia (CIN) or suspected cervical malignancy; visible HPV-related cervical lesions or genital warts; active pelvic inflammatory disease requiring systemic treatment; immunosuppressive disorders or ongoing immunosuppressive therapy; prior cervical surgery (e.g., conization or loop electrosurgical excision procedure); and incomplete follow-up data.

### 2.4 Baseline evaluation

All participants underwent standardized gynecological examination including speculum evaluation. Before assessment, cervical mucus and discharge were gently removed with a sterile cotton swab to optimize visualization.

The ectopy area was assessed visually and measured as the maximum diameter of columnar epithelium extending beyond the external cervical os (cm).

Baseline cervical cytology and HPV testing were performed to exclude premalignant or malignant pathology. When clinically indicated, microbiological evaluation for genital infections was completed; confirmed infections were treated before the study treatment was initiated.

Colposcopy-directed biopsy was not performed routinely in all participants; advanced diagnostic evaluation was reserved for clinically suspicious findings.

All clinical assessments were performed by a single experienced gynecologist (S.S.) to reduce inter-observer variability.

Because a single assessor performed all measurements, inter-rater reliability could not be calculated.

### 2.5 Treatment protocol

All enrolled patients received hyaluronic acid vaginal ovules (Cicatridine) once daily for 14 consecutive days. Patients were instructed to apply the ovule at bedtime to maximize mucosal contact.

### 2.6 Follow-up and outcomes

Clinical follow-up was performed at 4 weeks, 8 weeks, and 3 months after treatment completion. At each visit, speculum examination was repeated to evaluate epithelial transformation and residual ectopy.

The primary outcome was epithelial transformation, expressed as the percentage of baseline ectopic columnar epithelium replaced by squamous epithelium.

Epithelial transformation rate (%) was calculated as follows: ((baseline ectopy diameter − follow-up ectopy diameter)/baseline ectopy diameter) × 100. Because this calculation is based on maximum visible diameter rather than planimetric area, it should be interpreted as a pragmatic clinical estimate.

Secondary outcomes included descriptive evaluation of clinical symptoms (vaginal discharge, contact bleeding, dyspareunia, and pelvic pain).

### 2.7 Statistical analysis

Statistical analyses were performed using SPSS (version 25.0; IBM Corp., Armonk, NY, United States). Continuous variables are presented as mean ± standard deviation, and categorical variables as frequencies and percentages.

Changes in epithelial transformation over time were analyzed with repeated-measures analysis of variance (ANOVA). Sphericity assumptions were assessed and corrected when necessary. Bonferroni-adjusted post-hoc pairwise comparisons were used to compare time points.

A two-sided *p* < 0.05 was considered statistically significant. *A priori* sample size estimation with G*Power (version 3.1.9.6) assuming a moderate effect size (*f* = 0.25), *α* = 0.05, and power of 80% yielded a minimum sample size of 98 participants; therefore, the final sample of 121 was considered adequate.

## 3 Results

### 3.1 Baseline characteristics

A total of 121 women aged 24–41 years completed follow-up. No participants were excluded because of incomplete follow-up. Mean age was 32.5 ± 4.37 years. Baseline demographic and obstetric characteristics are shown in Table 1. Mean gravida was 2.67 ± 3.55, mean parity was 1.97 ± 0.94, and mean number of previous abortions was 0.25 ± 0.54. A history of previous dilatation and curettage was present in 16.5% of patients. All patients had normal cervical cytology and negative HPV test results at baseline.

**Table 1:** Baseline demographic and obstetric characteristics of the study population.

Variable	Value
Number of patients	121
Age (mean ± SD)	32.5 ± 4.37
Age range	24–41
Gravida (mean ± SD)	2.67 ± 3.55
Parity (mean ± SD)	1.97 ± 0.94
Abortus (mean ± SD)	0.25 ± 0.54
Previous dilatation & curettage	16.5%

Values are presented as mean ± standard deviation or percentage where appropriate. Gravida represents the total number of pregnancies, parity indicates the number of deliveries beyond the age of viability, and abortus represents the number of pregnancy losses before viability. Previous dilatation and curettage refers to a history of uterine evacuation procedures performed prior to study enrollment.

### 3.2 Cervical epithelial outcomes

At baseline, mean ectopy size was 1.95 ± 0.70 cm. Following hyaluronic acid therapy, progressive epithelial transformation was observed throughout follow-up.

Mean epithelial transformation increased from 61.5% ± 19.8 at 4 weeks to 74.6% ± 21.2 at 8 weeks and 82.9% ± 20.4 at 3 months (Table 2).

**Table 2:** Cervical epithelial transformation outcomes during follow-up after vaginal hyaluronic acid therapy (Cicatridine).

Outcome	Value
Baseline ectopy size (cm)	1.95 ± 0.70
Epithelial transformation rate at 4 weeks (%)	61.5 ± 19.8
Epithelial transformation rate at 8 weeks (%)	74.6 ± 21.2
Epithelial transformation rate at 3 months (%)	82.9 ± 20.4

Baseline ectopy size was measured as the maximum diameter of columnar epithelium extending beyond the external cervical os during speculum examination. Epithelial transformation rate represents the proportion of ectopic columnar epithelium replaced by squamous epithelium during follow-up visits. Clinical evaluations were performed at 4 weeks, 8 weeks, and 3 months after completion of treatment.

Repeated-measures ANOVA demonstrated significant change over time (*F* (2, 240) = 402.58, *p* < 0.001, partial η2 = 0.77). Post-hoc pairwise comparisons were significant between all follow-up time points (*p* < 0.001 for all).

**Figure 1:**
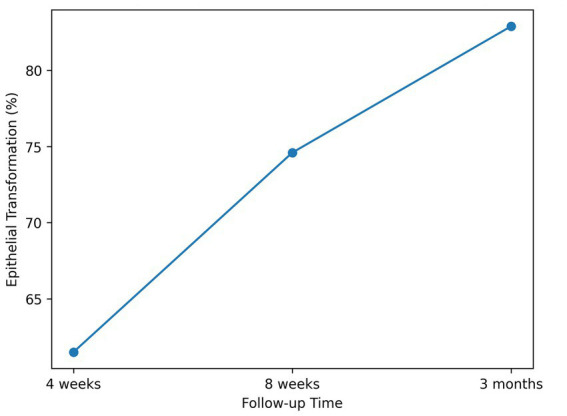
Temporal changes in epithelial transformation rates during follow-up.

The temporal pattern of epithelial transformation is illustrated in Figure 1.

### 3.3 Distribution of presenting symptoms

The distribution of baseline presenting symptoms is shown in Table 3. The most common symptom was abundant leukorrhea (66.1%), followed by contact bleeding (12.4%), dyspareunia (11.6%), chronic cervicitis (7.4%), and pelvic pain (2.5%).

**Table 3:** Distribution of presenting symptoms among patients with symptomatic cervical ectopy.

Symptom	*n*	Percentage
Abundant leukorrhea	80	65.3%
Contact bleeding	15	12.4%
Chronic cervicitis	9	6.6%
Dyspareunia	14	11.6%
Pelvic pain	3	2.5%

Symptoms were categorized according to the primary clinical complaint recorded at baseline. Abundant leukorrhea refers to persistent vaginal discharge associated with cervical ectopy. Contact bleeding indicates bleeding occurring during or after sexual intercourse. Chronic cervicitis represents recurrent inflammatory symptoms of the cervix. Dyspareunia refers to pain during sexual intercourse, while pelvic pain indicates chronic lower abdominal discomfort associated with cervical pathology.

**Figure 2:**
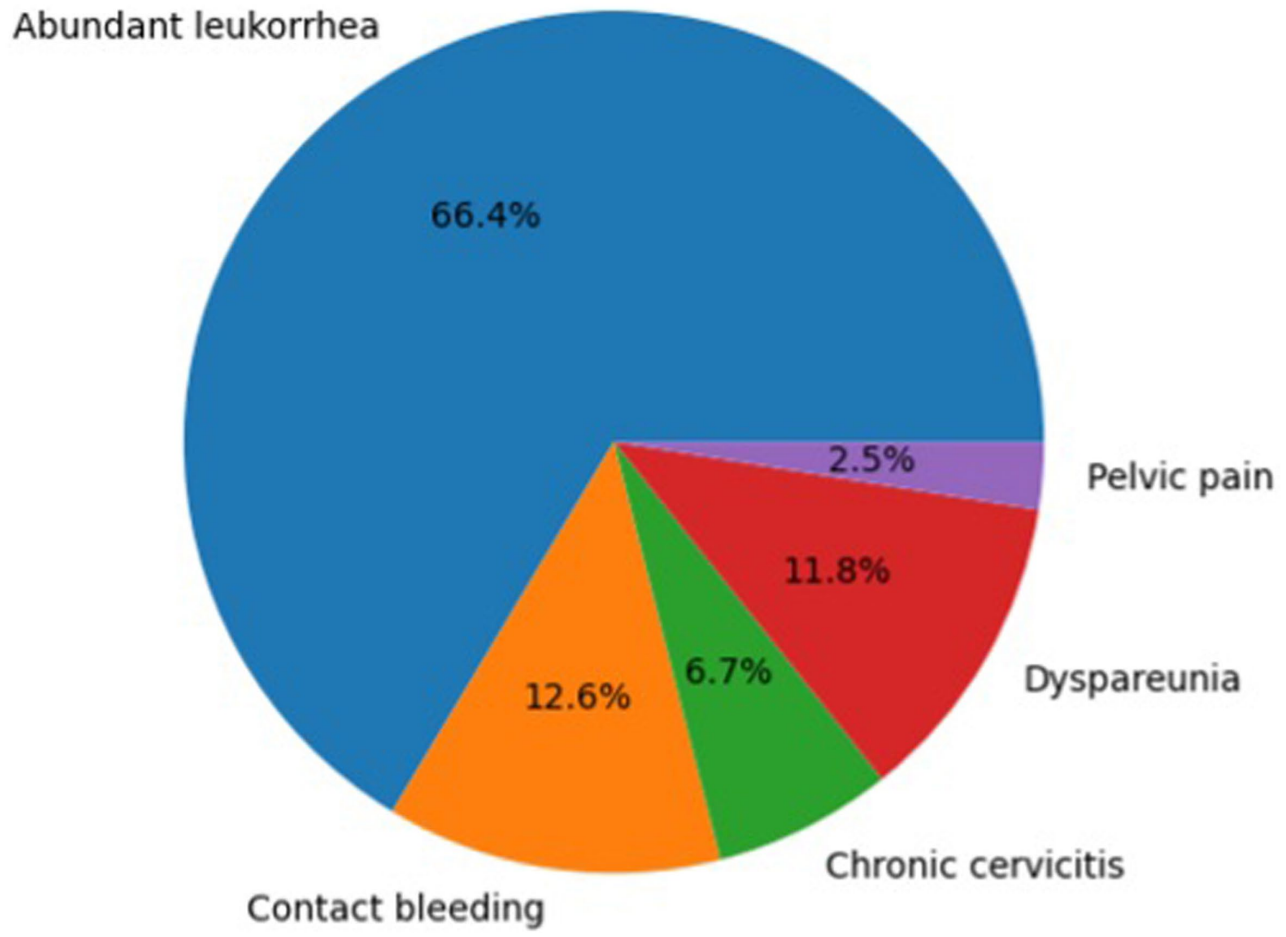
Distribution of presenting symptoms in patients with cervical ectopy.

The symptom distribution is also illustrated in Figure 2.

## 4 Discussion

In this prospective observational cohort, vaginal hyaluronic acid ovule therapy was associated with progressive epithelial transformation over follow-up. Transformation rates increased from 4 weeks to 8 weeks and reached the highest values at 3 months, suggesting a time-dependent mucosal remodeling pattern.

Our findings should be interpreted in the context of existing evidence, where ablative modalities—especially cryotherapy—have historically been used for symptomatic cervical ectopy (3, 4). Prior studies have reported symptom relief after cryotherapy and improvements in patient-centered outcomes, including sexual function and quality of life (3, 10, 11). At the same time, procedure-related adverse effects and potential cervical tissue consequences remain relevant considerations (5).

In this uncontrolled cohort, temporal improvement was observed; however, these data do not establish treatment efficacy. Conceptually, the observed cervical change is more appropriately interpreted as squamous metaplasia of exposed columnar epithelium rather than classical wound healing. Hyaluronic acid may influence the local cervicovaginal microenvironment (e.g., hydration and inflammatory modulation), but a direct causal mechanism for metaplastic transformation remains hypothetical (6, 7). Previous gynecologic reports on cervicovaginal use of hyaluronic acid provide supportive but non-comparative clinical context (8, 9).

Although squamous metaplasia is commonly a benign physiological process, excessive cervical remodeling and procedure-related tissue effects have been associated in some contexts with adverse outcomes such as stenosis or scarring (5). Therefore, future controlled studies should include pre- defined safety endpoints in addition to transformation metrics.

However, causality cannot be inferred from our results. Cervical ectopy may regress spontaneously, and the absence of a placebo or active comparator arm prevents direct attribution of epithelial changes to treatment effect. In addition, symptom evaluation was not based on a validated patient-reported scoring instrument (e.g., FSFI or SF-12); epithelial assessment relied on visual diameter-based clinical measurement; inter-rater reliability was not estimable because assessments were performed by a single examiner; and colposcopy-guided histopathologic confirmation was not available routinely in all participants.

Despite these limitations, the study provides descriptive longitudinal follow-up data and supports the feasibility of hypothesis generation for future comparative research.

Future research should prioritize randomized controlled designs comparing hyaluronic acid with placebo and with established ablative treatments (e.g., cryotherapy), while incorporating validated patient-reported instruments (e.g., FSFI pain-domain assessment and SF-12 quality-of-life scoring) and objective epithelial endpoints (10, 11). Such studies are required to define comparative effectiveness, durability of response, and optimal patient selection.

Overall, our findings are exploratory and hypothesis-generating, and should not be interpreted as evidence of clinical effectiveness.

## 5 Conclusion

In this prospective uncontrolled observational study, vaginal hyaluronic acid therapy was temporally associated with progressive epithelial transformation in symptomatic cervical ectopy.

Given the physiological nature of cervical ectopy and the possibility of spontaneous regression, the observed changes cannot be attributed solely to the intervention. Therefore, the current findings should be interpreted as exploratory.

Well-designed randomized controlled trials with placebo or active comparators, standardized patient-reported symptom instruments, and predefined safety endpoints are required before any efficacy claims can be made for this non-ablative approach.

## Data Availability

The original contributions presented in the study are included in the article/supplementary material, further inquiries can be directed to the corresponding author.
